# Frequency of Student Resource Use and Academic Performance in Preclerkship Education: A Survey Study

**DOI:** 10.1007/s40670-022-01674-y

**Published:** 2022-11-10

**Authors:** Uzoma Ikonne, Adrienne Brodie, Curt Bay, Anna Campbell

**Affiliations:** 1 Department of Physiological Sciences, Eastern Virginia Medical School, 700 W. Olney Rd, Norfolk, VA USA; 2 Fine Family Academy of Medical Educators, Eastern Virginia Medical School, P.O. Box 1980, Norfolk, VA USA; 3 A.T. Still Memorial Library, A.T. Still University, 5850 E. Still Circle, Mesa, AZ USA; 4 Department of Interdisciplinary Health Sciences, Arizona School of Health Sciences, A.T. Still University, 5850 E. Still Circle, Mesa, AZ USA; 5 Department of Anatomy, School of Osteopathic Medicine in Arizona, A.T. Still University, 5850 E. Still Circle, Mesa, AZ USA

**Keywords:** Resource use, External resources, Practice questions, Exam performance, Self-regulated learning

## Abstract

Medical students have unprecedented access to a large variety of learning resources, but patterns of resource use, differences in use across education cohorts, and the relationship between resource use and academic performance are unclear. Therefore, the purpose of the current study was to evaluate student resource use and its relationship to academic performance during preclerkship years. First-year and second-year medical students completed a 10-question electronic survey that assessed likelihood of using outside resources recommended by others, reasons for using outside resources, frequency of use of resources, and use of outside resources for specific disciplines. Outcomes were compared between the 2 cohorts of students. First-year students were more likely to use instructor-produced resources and self-generated study resources, and second-year students were more likely to use board review resources. Although differences were found between cohorts for frequency of use of certain resources, correlations between resource use and academic performance were modest. Overall, our results indicated that student use of study resources changed between the first and second years of medical school. These results suggest opportunities for medical educators to guide students in the selection and effective use of outside resources as they mature as self-regulated learners. Further, since students seem to extensively use external resources for learning, institutions should consider calibrating their curriculum and teaching methods to this learning style and providing high-quality, accessible resource materials for all students to reduce the potential impact of socioeconomic factors on student performance.

## Introduction

The landscape of medical education is shifting, and students have an unprecedented number of resources and information available to them. As such, many students are abandoning traditional didactic methods of in-person classroom learning, and the exigencies of the COVID-19 pandemic have accelerated these changes [1, 2]. Concurrently, the field of medical education is embracing the concept of self-regulated learning, whereby students develop personalized strategies for independent learning, such as selection of resources and use feedback to achieve desired academic outcomes [3–5]. Therefore, students need to learn how to select, critically evaluate, and effectively use information and resources from sources outside the classroom.

In addition to using resources provided by instructors in their academic courses, medical students typically include other resources as part of their study strategy. For example, they may use outside resources from the Internet, board preparation resources, and notes and charts from other students. Shershneva et al. [6] noted that effective use of resources was an important skill for medical training and practice. Given that students are attending in-person classes less frequently and considering the theoretical framework of self-regulated learning, it is important to understand how students use resources to inform us how they apply various learning strategies to achieve academic goals [1].

Research investigating resource use by medical students found a range of factors that influenced resource selection, such as ease of access and perceived quality of the information [7–9]. In a study of a single course [10], medical and dental student resource use did not vary significantly by learning resource type. In another study [11], the use of review books and Internet resources during a clerkship was significantly higher than the use of the recommended textbook. Other studies have reported positive correlations between resource use and exam performance [12, 13].

At the institution of the current study, osteopathic medical students progress through an integrated systems-based curriculum and distributed model, learning in community health centers during their second year. The curriculum of the first year is primarily lecture based (in-person and/or recorded) with active learning sessions. The students are primarily assessed with licensing exam style-like questions developed by the faculty. In courses, some instructors provided required or recommended readings and this is determined by the individual instructor. Although faculty have some awareness of student resource use through anecdotal interactions and feedback from advisees, patterns of resource use during preclerkship years, such as frequency of use of various resource types and reasons for use of outside resources, are unknown. Further, the relationship between resource use and academic performance is unclear. Therefore, the purpose of the current study was to evaluate student resource use and its relationship to academic performance during preclerkship years. A better understanding of student resource use and selection may be informative for instructors and result in improved curricular development and student advising.

## Materials and Methods

### Study Design and Participants

The current survey study was designed to determine the frequency of use of various study resources by first-year and second-year medical students and whether resource use was associated with academic performance. The study was conducted during the 2019–2020 academic year. An e-mail invitation with a link to an electronic survey was sent to first-year students in October 2019 and second-year students in January 2020. The survey instructions informed students about the study purpose and indicated that participation was voluntary with no direct benefit and that they could stop participating at any time. They were also informed that they would be required to provide identifying information in the survey so responses could be linked with academic records. Study investigators were blinded to identifying information, and student confidentiality was preserved during analysis and data reporting. The survey took about 15 min to complete. Students had 2 months to complete the survey and were sent 2 reminder e-mails. The A.T. Still University-Arizona Institutional Review Board approved this study #2019–116. Students provided their consent by completing the survey.

### Survey Design

An electronic survey (Qualtrics, Seattle, WA) with 10 questions was created specifically for the current study (Appendix 1). A group composed of faculty and students reviewed the survey for face and content validity. Four questions collected demographic information about name, age, sex, and highest degree attained before medical school matriculation. Four questions used Likert-type responses to assess likelihood of using outside resources (not recommended for class) recommended by others (e.g., instructors, classmates, and Internet), reasons for using outside resources (e.g., exam preparation, more detail, and practice questions), frequency of use of resources (instructor-produced resources, outside resources, student-generated resources), and use of outside resources for specific disciplines. Two open-ended questions asked about other reasons for using outside resources and for additional comments about the use of outside resources.

### Statistical Analysis

Survey responses were summarized using frequency and percentage. Mann–Whitney tests were used to compare Likert-type responses between first-year and second-year students. Spearman correlation coefficients were used to estimate the strength of associations between resource use and academic performance. For academic performance, data were collected from students’ academic records for course grades and from Comprehensive Osteopathic Medical Licensing Examination of the United States (COMLEX-USA) Level 1 scores for second-year students. Student grade point average (GPA) was used for analyses and was obtained and coded by a data informatics specialist with Family Educational Rights and Privacy Act authorization. The GPA data were considered separately and collectively for both years for analyses. Because of the large number of comparisons, a familywise error approach [14] was used to control for potential inflation of type I errors. Specifically, a Bonferroni correction was used, so the *α* level did not exceed .05 divided by the number of tests in each comparison. For all other analyses, an *α* of .05 (2-tailed) was used for statistical significance. SPSS version 26 (IBM Corp., Armonk, NY) was used for all data analyses.

## Results

### Participants

Eighty-eight (54.3%) of 162 first-year students responded to the survey, and 67 (62.6%) of 107 s-year students responded. Participants included 53 females (34.2%) and 46 males (29.7%); 56 (36.1%) students did not indicate their sex (Table 1).

**Table 1:** Demographic characteristics of first-year and second-year medical students participating in the current study

**Demographic characteristic**	**No. (%)**		
	**Total (*N* = 155)**	**First year (*n* = 88)**	**Second year (*n* = 67)**
Age, y			
20–25	81 (52.3)	51 (58.0)	30 (44.8)
26–30	65 (41.9)	33 (37.5)	32 (47.8)
Over 30	8 (5.2)	4 (4.5)	4 (5.9)
Sex			
Male	46 (29.7)	20 (22.7)	26 (38.8)
Female	53 (34.2)	30 (34.1)	23 (34.3)
Did not respond	56 (36.1)	38 (43.2)	18 (26.9)
Highest degree attained			
Bachelor of arts	28 (18.1)	17 (19.3)	11 (16.4)
Bachelor of science	98 (63.2)	58 (65.9)	40 (59.7)
Master of science	27 (17.4)	12 (13.6)	15 (22.4)
Doctoral degree	0 (0)	0 (0)	0 (0)
Other	2 (1.3)	1 (1.1)	1 (1.4)

Response numbers of survey questions varied because students did not answer all questions

### Use of Outside Resources Recommended by Others

 For survey items about outside resources recommended by others, responses of very likely and extremely likely were grouped as likely for analysis. The majority of first-year students indicated they were likely to use outside resources recommended by instructors (33/78, 42.3%), classmates (51/79, 64.5%), and the Internet (42/79, 53.1%) (Table 2). Conversely, a minority of second-year students indicated they were likely to use outside resources recommended by instructors (13/58, 22.4%), but a majority were likely to use those recommended by classmates (42/58, 72.4%) and the Internet (28/58, 48.3%). Data for use of outside resources for specific disciplines are presented in Appendix 2.

**Table 2:** Survey responses for use of outside resources recommended by others

**Other source**	**No. (%)**				
	**Not at all**	**Slightly**	**Somewhat**	**Very**	**Extremely**
Instructors					
First year (*n* = 78)	1 (1.3)	16 (20.5)	27 (34.6)	28 (35.9)	5 (6.4)
Second year (*n* = 58)	2 (3.4)	7 (12.1)	36 (62.1)	10 (17.2)	3 (5.2)
Classmates or other medical students					
First year (*n* = 79)	0 (0)	6 (7.6)	22 (27.8)	40 (50.6)	11 (13.9)
Second year (*n* = 58)	1 (1.7)	4 (6.9)	11 (19.0)	29 (50.0)	13 (22.4)
Internet resources (Google or Internet forum)					
First year (*n* = 79)	1 (1.3)	10 (12.7)	25 (31.6)	25 (31.6)	17 (21.5)
Second year (*n* = 58)	2 (3.4)	11 (19.0)	17 (29.3)	16 (27.6)	12 (20.7)
Library					
First year (*n* = 79)	12 (15.2)	26 (32.9)	27 (34.2)	13 (16.5)	1 (1.3)
Second year (*n* = 57)	16 (28.1)	20 (35.1)	17 (29.8)	4 (7.0)	0 (0)
Other					
First year (*n* = 58)	13 (22.4)	10 (17.2)	21 (36.2)	8 (13.8)	6 (10.3)
Second year (*n* = 48)	13 (27.1)	7 (14.6)	19 (39.6)	6 (12.5)	3 (6.3)

Response numbers of survey questions varied because students did not answer all questions

### Reasons for Using Outside Resources

**Fig. 1:**
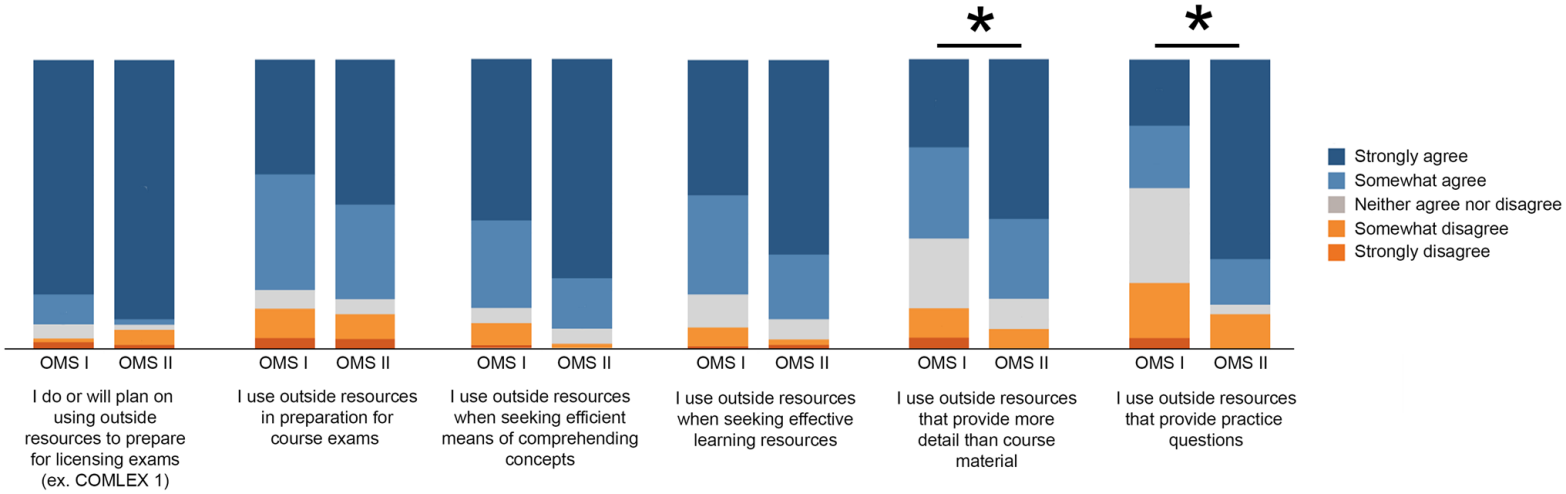
Bar graph of survey responses for reasons for using outside resources. All values are percentage endorsements within each category. *Both P < .008 based on an adjusted *α* of .008. Abbreviations: COMLEX 1, Comprehensive Osteopathic Medical Licensing Examination of the United States Level 1; OMS I, first-year osteopathic medical student; OMS II, second-year osteopathic medical student

Reasons for using outside resources are presented in Table 3; responses of somewhat agree and strongly agree were grouped for analysis. Most students indicated that they used outside resources for more detail (62.0% [49/79] first years, 82.8% [48/58] second years) and for practice questions (44.3% [35/79] first years, 84.5% [49/58] second years). Differences were found between cohorts for these 2 reasons (*P* = .002 and *P* = .001, respectively) (Fig. 1). No other differences were found between cohorts (all *P* > .05).

**Table 3:** Survey responses for reasons for using outside resources

**Reason for using outside resources**	**No. (%)**				
	**Strongly agree**	**Somewhat agree**	**Neither agree nor disagree**	**Somewhat disagree**	**Strongly disagree**
To prepare for licensing exams					
First year (*n* = 79)	64 (81.0)	8 (10.1)	4 (5.1)	1 (1.3)	2 (2.5)
Second year (*n* = 58)	52 (89.7)	1 (1.7)	1 (1.7)	3 (5.2)	1 (1.7)
To prepare for course exams					
First year (*n* = 78)	31 (39.7)	31 (39.7)	5 (6.4)	8 (10.3)	3 (3.8)
Second year (*n* = 58)	29 (50.0)	19 (32.8)	3 (5.2)	5 (8.6)	2 (3.4)
When seeking efficient means of comprehending concepts					
First year (*n* = 79)	44 (55.7)	24 (30.4)	4 (5.1)	6 (7.6)	1 (1.3)
Second year (*n* = 58)	44 (75.9)	10 (17.2)	3 (5.2)	1 (1.7)	0 (0)
When seeking effective learning resources					
First year (*n* = 79)	37 (46.8)	27 (34.2)	9 (11.4)	5 (6.3)	1 (1.3)
Second year (*n* = 58)	39 (67.2)	13 (22.4)	4 (6.9)	1 (1.7)	1 (1.7)
For more detail than provided by course material					
First year (*n* = 79)	24 (30.4)	25 (31.6)	19 (24.1)	8 (10.1)	3 (3.8)
Second year (*n* = 58)	32 (55.2)	16 (27.6)	6 (10.3)	4 (6.9)	0 (0)
For practice questions					
First year (*n* = 79)	18 (22.8)	17 (21.5)	26 (32.9)	15 (19.0)	3 (3.8)
Second year (*n* = 58)	40 (69.0)	9 (15.5)	2 (3.4)	7 (12.1)	0 (0)

Response numbers of survey questions varied because students did not answer all questions

### Frequency of Use of Resources

**Fig. 2:**
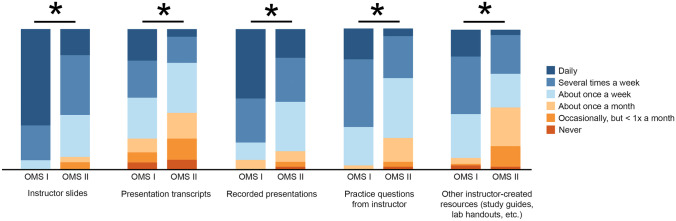
Bar graph of survey responses for frequency of use of instructor-produced resources. All values are percentage endorsements within each category. *All *P* < .01 based on an adjusted *α* of .01. Abbreviations: OMS I, first-year osteopathic medical student; OMS II, second-year osteopathic medical student

For survey items about frequency of use, responses of daily and several times a week were grouped as frequently for analysis. Data for use of instructor-produced resources are presented in Table 4. First-year students more frequently used instructor resources than second-year students. These included instructor slides, transcripts, recorded presentations, practice questions from instructors, and other instructor-created resources (*P* < .001, *P* = .002, *P* < .001, *P* < .001, and *P* < .001, respectively) (Fig. 2). First-year students used instructor slides (93.2% [68/73] vs 61.1% [33/54]), recorded presentations (80.8% [59/73] vs 51.9% [28/54]), and practice questions (69.8% [51/73] vs 35.2% [19/54]) more frequently than second-year students (Table 4). Frequency of instructor slide use was positively correlated with first-year GPA (*r*_s_ = .38, *P* = .002).

**Table 4:** Survey responses for frequency of use of instructor-produced resources

**Type of resource**	**No. (%)**					
	**Never**	**Occasionally, but less than once a month**	**About once a month**	**About once a week**	**Several times a week**	**Daily**
Instructor slides						
First year (*n* = 73)	0 (0)	0 (0)	0 (0)	5 (6.8)	18 (24.7)	50 (68.5)
Second year (*n* = 54)	0 (0)	3 (5.6)	2 (3.7)	16 (29.6)	23 (42.6)	10 (18.5)
ECHO recordings						
First year (*n* = 72)	1 (1.4)	6 (8.3)	4 (5.6)	18 (25.0)	23 (31.9)	20 (27.8)
Second year (*n* = 0)	0 (0)	0 (0)	0 (0)	0 (0)	0 (0)	0 (0)
Transcripts*						
First year (*n* = 72)	4 (5.6)	5 (6.9)	7 (9.7)	21 (29.2)	19 (26.4)	16 (22.2)
Second year (*n* = 54)	4 (7.4)	8 (14.8)	10 (18.5)	19 (35.2)	10 (18.5)	3 (5.6)
Recorded presentations						
First year (*n* = 73)	0 (0)	0 (0)	5 (6.8)	9 (12.3)	23 (31.5)	36 (49.3)
Second year (*n* = 54)	1 (1.9)	2 (3.7)	4 (7.4)	19 (35.2)	17 (31.5)	11 (20.4)
Practice questions from instructor						
First year (*n* = 73)	0 (0)	0 (0)	2 (2.7)	20 (27.4)	35 (47.9)	16 (21.9)
Second year (*n* = 54)	1 (1.9)	2 (3.7)	9 (16.7)	23 (42.6)	16 (29.6)	3 (5.6)
Assigned or recommended reading						
First year (*n* = 73)	8 (11.0)	23 (31.5)	15 (20.5)	20 (27.4)	7 (9.6)	0 (0)
Second year (*n* = 54)	17 (31.5)	17 (31.5)	10 (18.5)	9 (16.7)	1 (1.9)	0 (0)
Other instructor-created resources						
First year (*n* = 73)	2 (2.7)	1 (1.4)	3 (4.1)	23 (31.5)	30 (41.1)	14 (19.2)
Second year (*n* = 54)	1 (1.9)	8 (14.8)	15 (27.8)	13 (24.1)	15 (27.8)	2 (3.7)
Office hours or other instructor interactions						
First year (*n* = 73)	7 (9.6)	20 (27.4)	24 (32.9)	19 (26.0)	2 (2.7)	6 (8.2)
Second year (*n* = 54)	15 (27.8)	23 (42.6)	10 (18.5)	6 (11.1)	0 (0)	0 (0)
Instructor-led review sessions						
First year (*n* = 73)	9 (12.3)	12 (16.4)	18 (24.7)	21 (28.8)	7 (9.6)	6 (8.2)
Second year (*n* = 54)	6 (11.1)	16 (29.6)	21 (38.9)	7 (13.0)	5 (9.3)	2 (3.7)

Response numbers of survey questions varied because students did not answer all questions

*ECHO*, ECHO 360 lecture capture system

^*^Transcripts are written copy of the lecture narration

**Fig. 3:**
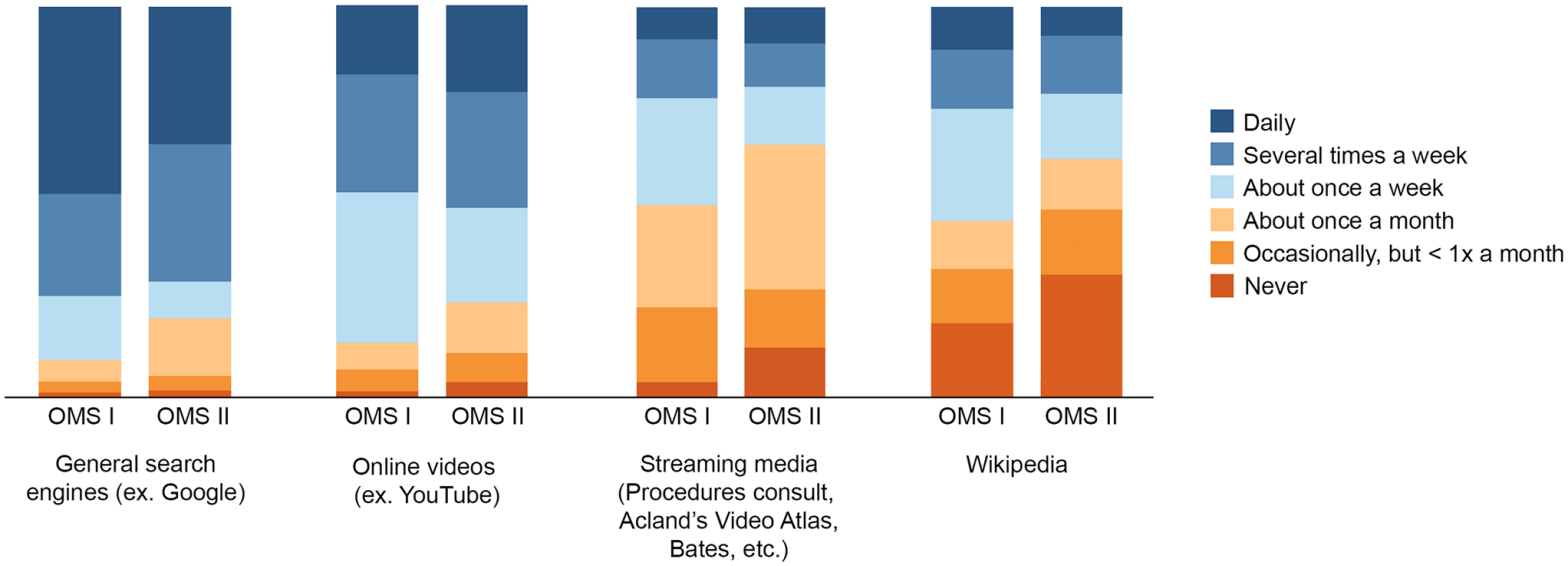
Bar graph of survey responses for frequency of use of general outside resources. All values are percentage endorsements within each category and no comparisons were significant. Abbreviations: OMS I, first-year osteopathic medical student; OMS II, second-year osteopathic medical student

**Fig. 4:**
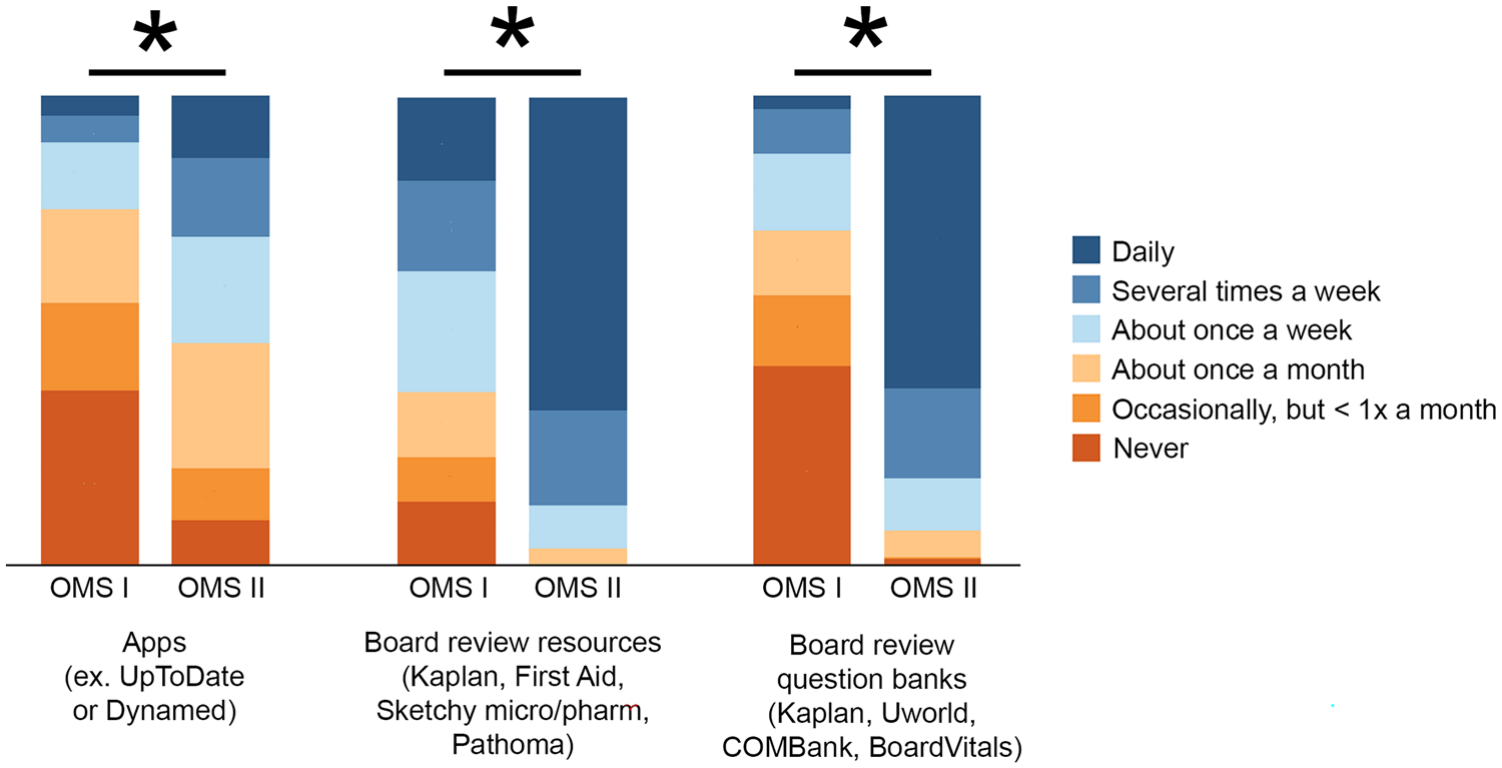
Bar graph of survey responses for frequency of use of specific outside resources related to board review. All values are percentage endorsements within each category. *All *P* < .02 based on an adjusted *α* of .02. Abbreviations: micro/pharm, microbiology/pharmacology; OMS I, first-year osteopathic medical student; OMS II, second-year osteopathic medical student

Regarding use of outside resources, the majority of students used search engines like Google (73.9% [54/73] first years, 70.4% [38/54] second years) and online videos like YouTube (47.9% [35/73] first years, 51.8% [38/54] second years) to help them study (Table 5 and Fig. 3). When considering both cohorts collectively, weak negative correlations were found between GPA and search engine (*r*_s_ =  −.20, *P* = .04) and Wikipedia (*r*_s_ =  −.19, *P* = .04) use. Second-year students used board review resources more often than first-year students (*P* < .001) (Fig. 4). Second-year students used board review resources such as First Aid (87.1% [47/54] vs 37.0% [27/73]), board review question banks (81.2% [43/53] vs 12.3% [9/73]), and apps such as UpToDate (30.2% [16/53] vs 10.0% [7/70]) more frequently than first-year students (all *P* < .02) (Table 5). When considering both cohorts collectively, modest positive correlations were found between GPA and use of board review resources (*r*_s_ = .31, *P* = .001) and board review question banks (*r*_s_ = .38, *P* < .001). Among first-year students, a modest negative correlation was found between GPA and use of board review question banks (*r*_s_ =  −.25, *P* = .047).

**Table 5:** Survey responses for frequency of use of outside resources

**Type of resource**	**No. (%)**					
	**Never**	**Occasionally, but less than once a month**	**About once a month**	**About once a week**	**Several times a week**	**Daily**
Supplemental textbooks or articles						
First year (*n* = 73)	21 (28.8)	16 (21.9)	9 (12.3)	20 (27.4)	6 (8.2)	1 (1.4)
Second year (*n* = 54)	12 (22.2)	13 (24.1)	8 (14.8)	10 (18.5)	11 (20.4)	0 (0)
General search engines						
First year (*n* = 73)	1 (1.4)	2 (2.7)	4 (5.5)	12 (16.4)	19 (26.0)	35 (47.9)
Second year (*n* = 54)	1 (1.9)	2 (3.7)	8 (14.8)	5 (9.3)	19 (35.2)	19 (35.2)
Literature database						
First year (*n* = 73)	21 (28.8)	11 (15.1)	17 (23.3)	15 (20.5)	5 (6.8)	4 (5.5)
Second year (*n* = 54)	12 (22.2)	11 (20.4)	13 (24.1)	8 (14.8)	8 (14.8)	2 (3.7)
Library website search						
First year (*n* = 73)	23 (31.5)	19 (26.0)	11 (15.1)	12 (16.4)	7 (9.6)	1 (1.4)
Second year (*n* = 54)	19 (35.2)	17 (31.5)	10 (18.5)	4 (7.4)	3 (5.6)	1 (1.9)
Online videos						
First year (*n* = 73)	1 (1.4)	4 (5.5)	5 (6.8)	28 (38.4)	22 (30.1)	13 (17.8)
Second year (*n* = 54)	2 (3.7)	4 (7.4)	7 (13.0)	13 (24.1)	16 (29.6)	12 (22.2)
Steaming media						
First year (*n* = 73)	3 (4.1)	14 (19.2)	19 (26.0)	20 (27.4)	11 (15.1)	6 (8.2)
Second year (*n* = 54)	7 (13.0)	8 (14.8)	20 (37.0)	8 (14.8)	6 (11.1)	5 (9.3)
Wikipedia						
First year (*n* = 73)	14 (19.2)	10 (13.7)	3 (4.1)	21 (28.8)	11 (15.1)	8 (11.0)
Second year (*n* = 54)	17 (31.5)	2 (3.7)	7 (13.0)	9 (16.7)	8 (14.8)	6 (11.1)
Apps						
First year (*n* = 70)	26 (27.1)	13 (18.6)	14 (20.0)	10 (14.3)	4 (5.7)	3 (4.3)
Second year (*n* = 53)	5 (9.4)	6 (11.3)	14 (26.4)	12 (22.6)	9 (17.0)	7 (13.2)
Board review resources						
First year (*n* = 73)	10 (13.7)	7 (9.6)	10 (13.7)	19 (26.0)	14 (19.2)	13 (17.8)
Second year (*n* = 54)	0 (0)	0 (0)	2 (3.7)	5 (9.3)	11 (20.4)	36 (66.7)
Practice questions from board review question banks						
First year (*n* = 73)	31 (42.5)	11 (15.1)	10 (13.7)	12 (16.4)	7 (9.6)	2 (2.7)
Second year (*n* = 53)	1 (1.9)	0 (0)	3 (5.7)	6 (11.3)	10 (18.9)	33 (62.3)
3D models						
First year (*n* = 73)	29 (39.7)	6 (8.2)	14 (19.2)	12 (16.4)	8 (11.0)	4 (5.5)
Second year (*n* = 54)	34 (63.0)	9 (16.7)	8 (14.8)	2 (3.7)	1 (1.9)	0 (0)

Response numbers of survey questions varied because students did not answer all questions

*3D*, 3-dimensional

**Fig. 5:**
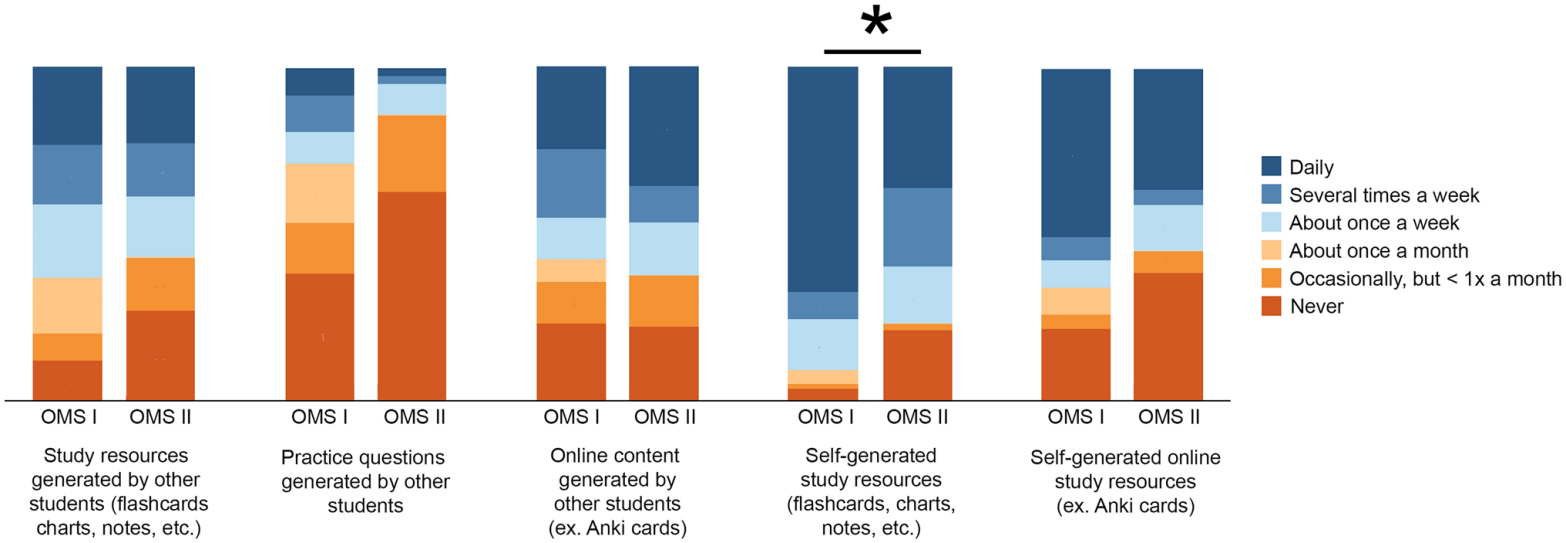
Bar graph of survey responses for frequency of use of student-generated resources. All values are percentage endorsements within each category. *P < .01 based on an adjusted *α* of .01. Abbreviations: OMS I, first-year osteopathic medical student; OMS II, second-year osteopathic medical student

Regarding use of student-generated resources, first-year students used self-generated study resources, such as flashcards or charts, more frequently than second-year students (75.3% [55/73] vs 51.9% [28/54], *P* < .01) (Table 6 and Fig. 5). When comparing frequency of the different resources used and COMLEX-USA Level 1 performance for second-year students, only 1 negatively correlated predictor was found between student use of outside student-generated resources and COMLEX-USA Level 1 performance (*r*_s_ =  −.32, *P* = .04). No other differences were found (all *P* > .05).

**Table 6:** Survey responses for frequency of use of student-generated resources

**Type of resource**	**No. (%)**					
	**Never**	**Occasionally, but less than once a month**	**About once a month**	**About once a week**	**Several times a week**	**Daily**
Study resources generated by other students						
First year (*n* = 73)	9 (12.3)	6 (8.2)	12 (16.4)	16 (21.9)	13 (17.8)	17 (23.3)
Second year (*n* = 54)	12 (22.2)	7 (13.0)	10 (18.5)	8 (14.8)	7 (13.0)	10 (18.5)
Practice questions generated by other students						
First year (*n* = 73)	28 (38.4)	11 (15.1)	13 (17.8)	7 (9.6)	8 (11.0)	6 (8.2)
Second year (*n* = 54)	27 (50.0)	10 (18.5)	11 (20.4)	4 (7.4)	1 (1.9)	1 (1.9)
Online content generated by other students						
First year (*n* = 73)	17 (23.3)	9 (12.3)	5 (6.8)	9 (12.3)	15 (20.5)	18 (24.7)
Second year (*n* = 54)	10 (18.5)	7 (13.0)	9 (16.7)	7 (13.0)	5 (9.3)	16 (29.6)
Self-generated study resources						
First year (*n* = 73)	3 (4.1)	1 (1.4)	3 (4.1)	11 (15.1)	6 (8.2)	49 (67.1)
Second year (*n* = 54)	10 (18.5)	1 (1.9)	7 (13.0)	8 (14.8)	11 (20.4)	17 (31.5)
Self-generated online study resources						
First year (*n* = 73)	16 (21.9)	3 (4.1)	6 (8.2)	6 (8.2)	5 (6.8)	37 (50.7)
Second year (*n* = 54)	17 (31.5)	3 (5.6)	10 (18.5)	6 (11.1)	2 (3.7)	16 (29.6)

Response numbers of survey questions varied because students did not answer all questions

### Open-ended Survey Responses

Twenty-seven student comments, 14 OMS I and 13 OMS II, were captured by the 2 open-ended survey questions. In response to the question about other reasons for using outside resources, a few students indicated that they thought instructor-produced lectures were insufficient for their learning needs. One first-year student wrote that they used other resources because they were “looking for condensed versions of the material. Lectures can sometimes feel disorganized.” A second-year student wrote, “In general, I found they are better at explaining important details. Some lecturers focus on the details too much and forget to teach the main concept.”

For the question asking for additional comments about use of outside resources, 2 students indicated that outside resources provided a way to supplement instructor-provided material. A first-year student wrote, “I prefer to get a very generalized view of a topic before watching lecture material. So oftentimes I will watch a youtube [sic] video prior to using course material.” A second-year student wrote, “I use outside resources to get better understanding of things that I did not fully understand from the lectures.” Other students considered the use of outside resources as the best way to prepare for board exams. One student wrote, “I enjoy our lectures, but sometimes feel that the high-yield board exam concepts are not always stressed in lecture. For that reason, I rely on outside resources to focus my studying to make sure I am fully understanding the material I will be heavily tested on come boards time!” Another wrote, “I cross-check everything against First Aid. If it’s not in First Aid, I generally don’t learn it.”

Conversely, some students indicated that they preferred interacting with the instructor and using instructor-produced resources. A first-year student wrote that they “don’t always use outside resources because coursework contains unique details that don’t comport with outside prep materials.” A second-year student wrote, “I find that if I don’t understand a concept, I would rather talk to the professor before looking at other outside resources.”

## Discussion

In the current study, we evaluated student resource use and its relationship to academic performance during preclerkship years. As expected, first-year and second-year medical students frequently used outside resources to study. Electronic resources, such as search engines and online videos, were used almost every day, and we found a modest negative correlation between search engine use and GPA. Further, second-year students were more likely to use outside board review resources than first-year students. Use of these resources showed modest positive correlations with GPA for both groups of students and weak negative correlations for first-year students only. First-year students were also more likely to use instructor-produced resources and self-generated study resources than second-year students. Overall, our results indicated that student use of study resources changes between the first and second years of medical school.

Our findings about frequency of use of outside resources, especially electronic resources, are consistent with previous studies [15, 16]. However, the weak negative correlations between GPA and frequency of use of general search engines (e.g., Google) and Wikipedia in the current study suggested that these resources may not be effective study tools. A possible explanation for the poorer academic performance of those using these outside resources may be related to the quality of the information. According to Choi-Lundberg et al. [9], students who more frequently rely on search engines or Wikipedia may find less accurate information than in primary resources. In another study, Judd and Elliott [17] found that first-year students frequently used Google and Wikipedia to study even though they did not rate the quality of the information as highly as other resources. Perhaps, students are using these outside electronic resources because they believe that e-learning resources are more efficient for locating and learning high-yield information [18]. Further, if instructor-produced resources are difficult to search, students may perceive that lack of efficiency as a barrier to their use. Therefore, faculty should consider the volume and organization of information when designing content for student use. This suggestion is supported by research that recommends faculty provide materials that maximize student learning [19]. Alternatively, experienced faculty and students could use their expertise to improve the content quality of existing external resources commonly used by students [20, 21].

In the current study, our finding that first-year students used instructor-produced resources more frequently than second-year students was expected. As the first-year students adapted to the challenges of medical school, it seems likely that they preferred to become familiar with the instructor-produced and institution-provided resources before seeking outside resources. Specifically, our first-year students indicated that they were more likely to use recorded presentations, practice questions, handouts, and transcripts to study and that they were less likely to use assigned or recommended readings, instructor-led review sessions, and office hours or other instructor interactions. In general, review sessions and office hours occur less frequently, which may explain their lower frequency of use. Further, less frequent use of assigned or recommended readings is consistent with other studies [22, 23]. Additional research suggests students are less motivated to use textbooks and instructor-suggested readings when electronic resources are available and are perceived as more efficient [15, 20].

Our study findings may also represent a shift in focus as students progress through their medical education, where second-year students become less reliant on instructor-produced resources. For example, our second-year students reported more frequent use of board review resources and board review question banks than first-year students. This finding is expected given that second-year students are required to take board exams at the end of the academic year. It is also consistent with responses from second-year students regarding their reasons for use of outside resources. Second-year students indicated they were more motivated to use outside resources to obtain additional detail, seek efficient and effective learning resources, and identify additional practice questions. Similar to our study, Scott et al. [15] also found students used digital resources for practice questions.

In the current study, we found that students used self-generated resources more frequently than student-generated resources. This finding probably reflects the practice of students focusing on the material they produce before using resources generated by their peers. We also found that self-generated and student-generated resources were used more frequently by first-year students than second-year students. Again, this finding likely reflects a shift in study focus as second-year students prepare for board exams and prioritize use of board review resources over individual or student-generated resources.

The more frequent use of outside resources by second-year students as they prepared for board exams was expected in the current study. Further, the negative correlation between the GPA of first-year students and frequency of use of outside practice questions and board review question banks suggested that an early focus on board preparation may distract students from other aspects of medical education that support academic performance. For instance, the use of board resources to study during the preclerkship curriculum may interfere with acquisition of foundational concepts. Further, board resources are typically based on review and should not be used to acquire new knowledge, which may explain the poorer academic performance. Since board review resources are not designed to teach new material, perhaps first-year students should be discouraged from using them. Although students who reported a higher use of question banks with multiple-choice questions had higher exam performance including on licensing exams [16, 24], the skills required for better exam performance may not be correlated with the skills needed to perform clinical responsibilities [13]. This finding suggests that the skills that enable an individual to perform well on a multiple-choice licensing examination may not correlate with the skills needed to perform as a competent physician.

The results of the current study may be especially important for educational environments where self-directed and independent learning are incorporated into the curriculum. Further, academic libraries may also find our results useful when considering new additions to the library collection. Since our study suggested that students frequently used electronic study resources, libraries should consider prioritizing additional online resources over print resources. For instance, review resources, exam preparation resources, or videos could be added to collections instead of new eBooks and journal subscriptions.

The results of the current study should be interpreted within the context of several limitations. Since it was conducted at a single institution, results may not be widely generalizable. Further, the current study only measured the frequency of resource use; thus, other considerations, such as how resources are used, were not measured. Another limitation may be related to self-selection bias. Because the survey was distributed electronically, students who were more likely to use electronic outside resources may have been more likely to complete our survey. In addition, this study relies on self-reported student data which may not match actual student resource use.

Our results highlight the need for faculty to intentionally design learning sessions and curricula that promote the maturity of their students as self-regulated learners. Because access to numerous high-quality external resources, such as question banks and review videos, may be cost prohibitive to students from lower socioeconomic households, our observed preference for use of outside resources to study may exacerbate inequalities that exist among medical students [25]. Therefore, it is incumbent on faculty and institutions to produce and provide high-quality resources that are accessible to all students. Further, faculty and institutions should guide students on how to select and evaluate study resources that allow them to proficiently use learning resources as they develop as self-regulated learners.

## Conclusion

Results of the current study suggested that patterns of resource use by first-year and second-year medical students differed across the preclerkship years. Further, the frequency of resource use was not, in general, correlated with academic performance. Overall, our study may advance opportunities for medical educators to guide students in the selection and use outside resources as they mature as self-regulated learners. As external resources become more abundant and easily accessible to medical students, institutions should consider their role in the development of their students as self-regulated learners and self-directed clinicians.

## Data Availability

All data generated or analyzed during the current study are included in this published article and its supplementary information files.

## Appendix 1. Student Resource Use Survey

Survey Participation Information

The attached survey is for research conducted by the School of Osteopathic Medicine in Arizona (SOMA) at A. T. Still University. The purpose of this study is to increase understanding of student resource use and its relationship to academic performance. Your participation is voluntary and will only involve completing the questionnaire. Completing this questionnaire should require 15 min of your time. Please do not use any outside assistance while answering these questions. Please answer each question honestly and to the best of your ability. You will be asked to identify yourself on the questionnaire, which will allow survey responses to be linked with your academic records. All information abstracted from your academic records including course grades and discipline scores as well as this survey, will be coded by a Family Educational Rights and Privacy Act (FERPA)-trained individual to remove names and other identifying information before transferring the data to the principal investigators. No names or other identifying information will be used in discussing or reporting data. The results of this research might be published. Any research reports or publications resulting from this research will not reveal your name or identity. All of your data will remain confidential. There are no direct benefits to you for completing this questionnaire. By completing the questionnaire, you agree to allow your survey responses and academic records to be used for this study. Your participation is voluntary, and you may refuse to complete the questionnaire or stop at any time if you wish.

**Please indicate which of the following categories most closely approximates the frequency of your use of each of the following resources. (Answer in table below)**

**Table:**

	Never	Occasionally, but less than once a month	About once a month	About once a week	Several times a week	Daily
Instructor slides						
ECHO recordings						
Transcripts						
Recorded presentations						
Practice questions provided by the instructor						
Assigned or recommended reading (textbook or articles)						
Other instructor created resources (study guides, lab handouts, etc.)						
Office hours or other instructor interactions						
Instructor Led Review sessions						
Supplemental textbooks or articles (not assigned by the instructor)						
General search engines (ex. Google)						
Online videos (ex. YouTube)						
Wikipedia						
Board review resources (Kaplan, First Aid, Sketchy micro/pharm, Pathoma)						
Practice Questions provided Board review question banks (Kaplan, Uworld, COMBank)						
Review/study sessions with other students (not instructor-led)						
Study resources (ex. flashcards, charts, or notes) generated by other students						
Practice questions generated by other students						
Online content generated by other students (ex. Anki cards)						
Self-generated study resources (flashcards, charts, notes, etc.)						
Self-generated online study resources (ex. Anki cards)						

**How often do you use outside resources for the following disciplines? (Answer in table below).**

**Table:**

	Never	Occasionally	About 1 × a month	About 1x/week	Several times/week	Daily
Anatomy						
Embryology						
Biochemistry						
Clinical Sciences						
Genetics/Molecular Biology						
Osteopathic Principles and Practice						
Microbiology/Immunology						
Pathology						
Physiology						
Pharmacology						

Please provide any additional comments with regards to use of outside resources

## Appendix 2. Survey responses for use of outside resources for specific disciplines

**Table:**

**Specific discipline**	**No. (%)**					
	**Never**	**Occasionally, but less than once a month**	**About once a month**	**About once a week**	**Several times a week**	**Daily**
Anatomy						
First year (*n* = 72)	2 (2.8)	2 (2.8)	3 (4.2)	17 (23.6)	38 (52.8)	10 (13.9)
Second year (*n* = 53)	2 (3.8)	7 (13.2)	19 (35.8)	9 (17.0)	11 (20.8)	5 (9.4)
Embryology						
First year (*n* = 72)	23 (31.9)	5 (6.9)	17 (23.6)	13 (18.1)	8 (11.1)	6 (8.3)
Second year (*n* = 53)	11 (20.8)	9 (17.0)	17 (32.1)	7 (13.2)	5 (9.4)	4 (7.5)
Biochemistry						
First year (*n* = 72)	15 (20.8)	10 (13.9)	9 (12.5)	17 (23.6)	14 (19.4)	7 (9.7)
Second year (*n* = 53)	3 (5.7)	6 (11.3)	13 (24.5)	14 (26.4)	9 (17.0)	8 (15.1)
Clinical sciences						
First year (*n* = 71)	8 (11.3)	12 (16.9)	15 (21.1)	16 (22.5)	13 (18.3)	7 (9.9)
Second year (*n* = 54)	2 (3.8)	3 (5.7)	10 (18.9)	16 (30.2)	12 (22.6)	10 (18.9)
Genetics/molecular biology						
First year (*n* = 72)	8 (11.1)	11 (15.3)	15 (20.8)	20 (27.8)	11 (15.3)	7 (9.7)
Second year (*n* = 53)	8 (15.1)	6 (11.3)	7 (13.2)	19 (35.8)	9 (17.0)	4 (7.5)
Osteopathic principles and practice						
First year (*n* = 72)	34 (47.2)	9 (12.5)	12 (16.7)	10 (13.9)	4 (5.6)	3 (4.2)
Second year (*n* = 53)	17 (32.1)	15 (28.3)	5 (9.4)	6 (11.3)	7 (13.2)	3 (5.7)
Microbiology/immunology						
First years (*n* = 71)	4 (5.6)	4 (5.6)	8 (11.3)	16 (22.5)	30 (42.3)	9 (12.7)
Second years (*n* = 53)	1 (1.9)	3 (5.7)	3 (5.7)	8 (15.1)	24 (45.3)	14 (26.4)
Pathology						
First year (*n* = 72)	9 (12.5)	7 (9.7)	9 (12.5)	14 (19.4)	24 (33.3)	9 (12.5)
Second year (*n* = 53)	1 (1.9)	2 (3.8)	2 (3.8)	6 (11.3)	27 (50.9)	15 (28.3)
Physiology						
First year (*n* = 72)	9 (12.5)	5 (6.9)	7 (9.7)	20 (27.8)	21 (29.2)	10 (13.9)
Second year (*n* = 53)	1 (1.9)	4 (7.5)	7 (13.2)	15 (28.3)	17 (32.1)	9 (17.0)
Pharmacology						
First year (*n* = 72)	10 (13.9)	7 (9.7)	9 (12.5)	16 (22.2)	20 (27.8)	10 (13.9)
Second year (*n* = 52)	3 (5.8)	3 (5.8)	6 (11.5)	8 (15.4)	21 (40.4)	11 (21.2)

Response numbers of survey questions varied because students did not answer all questions
